# Modeling Early Stages of Bone and Joint Infections Dynamics in Humans: A Multi-Agent, Multi-System Based Model

**DOI:** 10.3389/fmolb.2020.00026

**Published:** 2020-03-12

**Authors:** Salma Alsassa, Thomas Lefèvre, Vincent Laugier, Eric Stindel, Séverine Ansart

**Affiliations:** ^1^Laboratory of Medical Information Processing (LaTIM - UMR 1101 INSERM), IBRS, Université de Bretagne Occidentale, Department of Medicine, Brest, France; ^2^Tekliko SARL, Paris, France; ^3^Iris UMR 8156 CNRS - U997 Inserm - EHESS - UP 13, Paris, France; ^4^AP-HP, Jean Verdier Teaching Hospital, Department of Legal and Social Medicine, Bondy, France; ^5^La Cavale Blanche University Hospital, Infection Diseases Unit, Brest, France

**Keywords:** bone and joint infections, agent-based model, NetLogo, multi-scale, bone remodeling, diagnosis, Staphylococcus aureus

## Abstract

Diagnosis and management of bone and joint infections (BJI) is a challenging task. The high intra and inter patient’s variability in terms of clinical presentation makes it impossible to rely on a systematic description or classical statistical analysis for its diagnosis. Advances can be achieved through a better understanding of the system behavior that results from the interactions between the components at a micro-scale level, which is difficult to mastered using traditional methods. Multiple studies from the literature report factors and interactions that affect the dynamics of the BJI system. The objectives of this study were (i) to perform a systematic review to identify relevant interactions between agents (cells, pathogens) and parameters values that characterize agents and interactions, and (ii) to develop a two dimensional computational model of the BJI system based on the results of the systematic review. The model would simulate the behavior resulting from the interactions on the cellular and molecular levels to explore the BJI dynamics, using an agent-based modeling approach. The BJI system’s response to different microbial inoculum levels was simulated. The model succeeded in mimicking the dynamics of bacteria, the innate immune cells, and the bone mass during the first stage of infection and for different inoculum levels in a consistent manner. The simulation displayed the destruction in bone tissue as a result of the alteration in bone remodeling process during the infection. The model was used to generate different patterns of system behaviors that could be analyzed in further steps. Simulations results suggested evidence for the existence of latent infections. Finally, we presented a way to analyze and synthesize massive simulated data in a concise and comprehensive manner based on the semi-supervised identification of ordinary differential equations (ODE) systems. It allows to use the known framework for temporal and structural ODE analyses and therefore summarize the whole simulated system dynamical behavior. This first model is intended to be validated by *in vivo* or *in vitro* data and expected to generate hypotheses to be challenged by real data. Step by step, it can be modified and complexified based on the test/validation iteration cycles.

## Introduction

Bone and joint infections (BJI) are challenging pathologies that seriously affect public health, by means of irreversible bone loss, functional impairment and reduced quality of life (Stengel et al., 2001). BJI can result from hematogenous spread of infection and develop within 2 weeks (acute hematogenous BJI) (Carek et al., 2001; Jorge et al., 2010). In adults, BJI develop more frequently from direct inoculation of bacteria secondary to trauma, internal fixation of a fracture, or prostheses placement and progress slowly within months (Smith et al., 2006; Birt et al., 2017). The incidence of BJI is increasing annually in the developed countries due to several reasons such as the rise in arthroplasties, and diabetes (Kurtz et al., 2011; Walter et al., 2012). In 2013, 70 cases per 100,000 of the population were reported as BJI related in France, compared to 54 cases in 2008, along with higher readmission frequency and longer hospital stays. Furthermore, the BJI inpatient mortality rate was reported as 4.6% (Grammatico-Guillon et al., 2012; Laurent et al., 2016). In the USA, Olmsted County, the BJI incidence increased from 11.4 cases per 100,000 of population per year in the period from 1969 to 1979 to 24.4 per year in the period from 2000 to 2009 (Kremers et al., 2015). BJI are also a significant cause of higher healthcare costs each year. The higher cost is due to prolonged and repeated hospitalizations supplemented by complex and long-term treatments, in addition to the need for surgical intervention (Gentry, 1997; Lipsky et al., 2007).

BJI are characterized by a complex biological system on both structural and functional levels. They are defined by multiple variables that have impacts on their pathogenesis (Ciampolini and Harding, 2000). They have a dynamic environment that comprised of different bone matrix qualities, cell types, in addition to the potential presence of biofilms or prostheses (Buckwalter et al., 1996). They encompass complex interactions at both cellular and molecular levels that are difficult to observe and control for using the traditional methods (Lebeaux et al., 2013). Thus, BJI should be better understood through investigating the system behavior that evolves from the interactions between the individual agents. Improving the understanding of the infection dynamics is key to selecting the optimum treatment method.

Numerous animal models have been proposed to study the BJI caused by *Staphylococcus aureus*, the most common cause of this disease (Crémieux and Carbon, 1997; Xing et al., 2012; Johansen et al., 2013). However, these models were limited by the duration of the experiments and the incapacity to observe and control for the interactions between the agents within the process (Mader, 1985; Lebeaux et al., 2013). In addition, the high inter and intra patient’s variability of the disease presentation imposes another limitation in front of the classical statistical analysis tools. Thus, the lack of the available evidence and the challenges that accompany the proposed traditional methods necessitate exploring the strength of computational tools to (1) simulate the BJI systems, (2) analyze their progression, and (3) provide insights in selecting evidence-based treatment strategies.

BJI development is the result of the continued interplay between bacteria and two systems within the host: the bone tissue and the immune defense system. Bacterial invasion of the bone leads to a cascade of adverse changes in bone tissue system components and functions. Specifically, it causes an imbalance in the bone remodeling process, through interactions between the bacteria and bone cells, leading to the activation of bone destruction pathway (Claro et al., 2011; Josse et al., 2015). Concurrently, the presence of bacteria triggers the innate immune cells to signal a response against the invader. The infected bone cells also take part in releasing cytokines that contribute to either directing immune cell reactions or increasing severe inflammatory damage (Bost et al., 1999; Marriott et al., 2005).

Having an integral insight into the disease needs to integrate the various components that manage and direct the dynamic interactions within the BJI process in a computational modeling framework. To date, scarce attempts in the literature investigated the BJI using computational modeling techniques. Pietro and associates (Lio et al., 2012), proposed a computational model to compare the effects of osteomyelitis and osteoporosis on bone remodeling process using the differential equations and probabilistic verification methods based on population-based approach. However, the model emerges from the equation-based approach which lacks the spatial distribution and the micro-interactions of the components, and it ignores the interplay with the immune system.

In this context, the agent-based modeling approach offers a promising framework to translate the system biology and generate a plausible representation of the individual component behavior. ABM approach is a rule-based, bottom-up approach where the system is represented through its entities (agents) that act autonomously in the environment, and the overall behavior of the system results from the accumulation of the local attitudes and interactions of the concerned agents. This approach is based on the principle that mainly local interactions are described. These interactions can take place between agents or between agents and their environment. Global behavior is therefore observed as a phenomenon emerging from the multiple interactions defined in the model. The simulation itself then makes it possible to observe the evolution of the system, by letting it “live” according to the rules and interactions that have been defined on a local scale (An et al., 2009; Shi et al., 2014).

The different features of the ABM approach such as the built-in randomness, spatial architecture, emergent behavior, and taking parallel mechanisms outcomes capability make ABM well suited to represent the biological system and display its hidden and multi-scale behavior (Folcik et al., 2007; Politopoulos, 2007). The ABM models could reproduce the complex behavior of the biological system with heterogeneous components and diverse essential rules, even if they are simple and not complete (Gelfand, 2013; Hammond, 2015). The ABM also has an intuitive paradigm that considers the individual character, decision and propensity. It has the ability to represent the non-linear relationships at the micro-level, which contributes to enhancing the understanding and controlling the different patterns of the system behavior (Kitano,2002a, b; Ghosh et al., 2011; Hernán, 2015). Using ABM facilitates system simulations to analyze processes that were not measured in the experimental set-up otherwise (Holcombe et al., 2012; Horn et al., 2012).

In this study, we identified experimental evidence on various aspects of BJI in adults following a literature review and integrated it in a multi-agent, multi-system model based synthesis. We focused on the early stage of the infection to investigate the critical role of innate immune cells in eliminating the bacteria, as well as the loss in bone tissue in this stage. We are focusing on how the cellular mechanisms and interactions influence the BJI development and give rise to the system-level behavior. We developed a two-dimensional agent-based model of BJI that introduces a plausible representation of the system and a simulated experimental environment to reproduce the infection and examine its dynamics as a result of cellular interactions. The BJI system will be modeled through the interaction between the components of three subsystems: the bacteria, the infected bone, and the immune response. Concerning the investigated bacteria, we studied here in this first model *S. aureus* in general, which is the major bacteria in BJI, notably because it expresses different strategies of persistence such as producing biofilm or hiding intracellularly, and for that it is mainly involved in relapsing infections. The model simulations will be used to test several hypotheses that exist in the literature and to investigate the effect of different parameters changes on the cells dynamics, such as the initial concentration of bacteria, which is a major element in the clinical expression of this infection, and the quality of the immune system during the first stage of infection.

## Materials And Methods

In this study, a three-step approach was followed: (i) to conduct a systematic literature search identifying behavior and interactions between agents in both cellular and molecular levels, as well as the values of the parameters involved, (ii) to develop a two-dimensional model of early stages of BJI using the ABM approach, based on the results obtained in the first step, and (iii)to conduct simulations to investigate the outcomes of the infected system under different conditions.

### Literature Review for Agents Interactions and Parameters Values Identification

The first step toward ABM model development was describing the BJI system and its behaviors and characterizing its components and their roles. A systematic literature search was conducted to retrieve the agents’ behaviors, rules, and interactions, and to identify the relevant parameters and their ranges of applicable values. By giving some parameters a range of values, we enriched the ABM model with the capacity of simulating variable behaviors of the agents and testing several hypotheses. We used the information from the basic biology to characterize the cellular and molecular attitudes of different components of BJI biological system during the first stage of infection; namely those cover the innate immune response, the bone remodeling cells and signals, and the bacteria development. These biological characteristics will be simplified and formulated as agents’ rules. The different parameters were retrieved by first looking for human studies, but regarding the lack of several information, we extended our search to include general characteristics of the components, animal models of BJI, and mathematical models of the bone remodeling process.

Search was conducted in PubMed and ScienceDirect databases exploring the relevant journal papers from their inception to date, July 2017. Literature search strategy, i.e., search queries and inclusion/exclusion criteria, are described in detail in Supplementary Material and Supplementary Table S1.

### The Agent-Based Model of Early Stages Bone and Joint Infections

#### The Environment

NetLogo was chosen for implementing the BJI model for its several features such as supporting investigating the agent behaviors over time and space and emerging the agents’ heterogeneity within its framework (Wilensky, 1999). Among ABM toolkits, NetLogo introduces an academic and community-supported tool to simulate the compound phenomena using a high-level multi-agent programming language and a robust, simple modeling platform.

**FIGURE 1:**
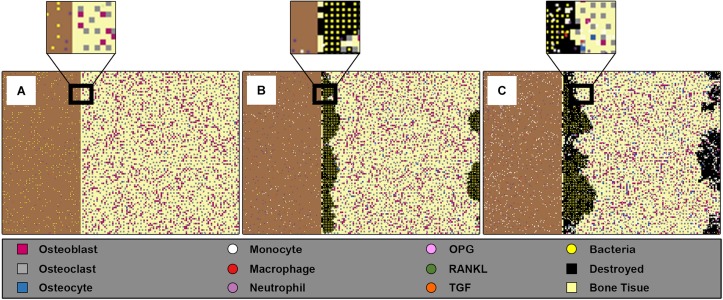
Screenshots of the ABM space at three different time steps for inoculum infection state of (5 × 10^2^CFU/mm^2^). The left rectangle in each sub-figure represents the bacteria population, and the right rectangle represents 2 mm^2^ of bone tissue where the bone cells, osteoblasts, osteoclasts, and osteocytes, are randomly allocated respecting their percentage and minimum distance. **(A)** Shows the initial state of the model at = 0 h, where the bacteria are randomly distributed in the adjacent surface, the left rectangle, with low presence of immune cells especially macrophages. **(B)** Shows the state at time *t* = 60 h, where the bacteria entered the bone tissue and started destroying it. **(C)** Shows the model’ state at *t* = 150 h, where the damage happens to the bone tissue, the black patches within bone tissue reflect this destruction, while at the same time the bacteria count was decreased because of engulfing by immune cells.

The general 2D space of our model was divided into two rectangles of overall dimension 151 × 101 patches (patches represent the grids in the landscape in NetLogo). The first rectangle (101 × 101 patches) was a bone tissue represented as a two-dimensional layer; we assumed this dimension according to 2 mm^2^ of bone tissue. We also assumed that this surface contained one basic multicellular unit (BMU). The second rectangle was the adjacent surface (50 × 101 patches), where the bacteria was initially present (Figure 1A). The virtual time scale was used in the environment “ticks” with each tick mapping to 1 h of the real-time. The model algorithm started with an initial state with all the parameters set to their initial values, followed by a set of rules and functions that were repeated until the end of the simulation.

#### Agent Types, Rules, and Parameters

**FIGURE 2:**
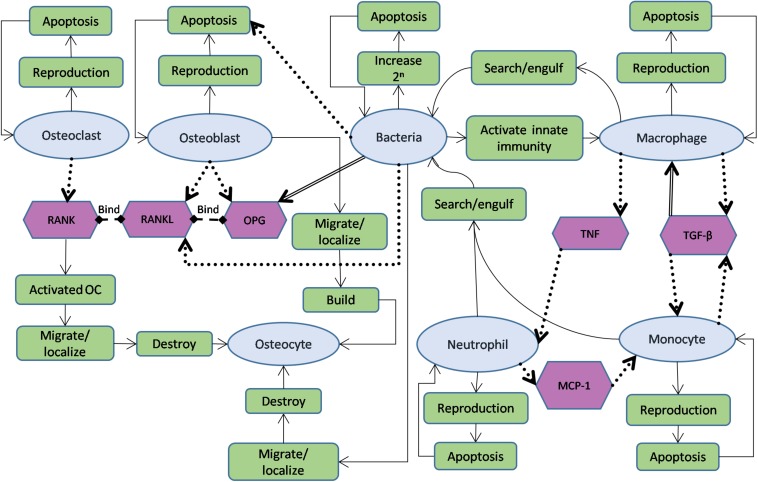
Schematic diagram showing agents used in the model, interactions between them, and governing functions for each of them. The oval shapes represent cell agents; hexagons represent signals in the model. The rectangle boxes represent the main functions and roles of each agent. A solid arrow indicates the flow of agent functions, a dotted arrow characterizes stimulation from source to target (destination), while a double-lined arrow reflects the opposite effect (source leads to reduce the destination object).

The agents in the model were chosen based on their importance during the first stage of the infection. The bone and immune cell agents interact with the bacteria agents through main interactions while maintaining their functions (Figure 2). Furthermore, the agents in the model and the mediators with their functions and roles are summarized in Tables 1, 2.

**TABLE 1:** List of the agents in the bone and joint infections agent-based model, their rules, and behaviors.

Agent type	Agent parameters	Agent’ rules in bond and joint infections ABM	References
Bacteria	Inoculum size Reproduction rate	Increase rapidly and spread spatially to invade the bone tissue, stimulate releasing RANKL and activating OC, stimulate immune defense and are engulfed by them, stimulate OB death	Fullilove et al., 2000; Claro et al., 2011; Olson and Horswill, 2013
Neutrophils (PMN)	Count, Lifespan Reproduction rate	Undergo reproduction and death function, recruited due to the presence of bacteria and try to ingest them, recruit MDM	Cassatella, 1995; Kaplanski et al., 2003; Bekkering, 2013; Boff et al., 2018
Macrophages (MA)	Count, Lifespan Reproduction rate	Undertake reproduction and death function, stimulated by the presence of bacteria and attack them, regulate macrophages and MDM recruitment through TGF-beta, stimulating neutrophils	Kim and Luster, 2015; Kaufmann and Dorhoi, 2016; Prame Kumar et al., 2018
Monocytes (MDM)	Count, Lifespan Reproduction rate	Undergo reproduction and death function, stimulated by the presence of bacteria, PMN, and MA after T hours, phagocytosis the bacteria, release TGF-beta to regulate macrophages and MDM recruitment	Deshmane et al., 2009; Lee et al., 2011; Gong et al., 2012; Ginhoux and Jung, 2014; Patel et al., 2017
Osteoblasts (OB)	Count, Lifespan Reproduction rate	Go through reproduction and death cycle, spatial localization, releasing RANKL and OPG, take a role in bone remodeling process: form new osteocytes	Jilka et al., 1998; Boyce and Xing, 2008; Shapiro, 2008
Osteoclasts (OC)	Count, Lifespan Reproduction rate	Go through reproduction and death cycle, spatial localization, bind with RANKL to be activated, take a role in bone remodeling process: destroying osteocytes	Parfitt, 1994; Blair, 1998; Eriksen, 2010
Osteocytes (OS)	Count, percentage	Form bone osteocytes cells network with respecting the minimum distance between them, derived from mature osteoblasts, destroyed by active osteoclasts	Franz-Odendaal et al., 2006; Bonewald, 2007; Schneider et al., 2010

**PMN, Neutrophils; MA, Macrophages; MDM, Monocyte-derived Macrophages; TGF-β, Transforming growth factor-beta; RANKL, Receptor activator of nuclear factor-kappa-B ligand; OPG, Osteoprotegerin.**

**TABLE 2:** List of the mediators and their effects that are represented in the bone and joint infections ABM.

Mediator variable	Mediator parameter	Source	Role in BJI agent-based model	References
RANKL	Concentration	Osteoblasts	Diffusion, activate osteoclasts by binding to them or inhibit their activation by binding with OPG	Anandarajah, 2009
OPG	Concentration	Osteoblasts	Diffusion, bind with RANKL to inhibit activating osteoclasts	Boyce and Xing, 2008
TGF-beta	Concentration	Macrophages Monocytes	Released by both monocyte and macrophages to increase monocytes recruitment and decrease macrophage recruitment	Ashcroft, 1999
MCP-1	Concentration	Neutrophils	Released by neutrophils to stimulate monocytes recruitment	Gopalakrishnan et al., 2013; Tecchio et al., 2014
TNF	Concentration	Macrophages	Released by macrophages to enlist neutrophils to the site	Prame Kumar et al., 2018

**BJI, bone and joint infections; TGF-β, Transforming growth factor-beta; RANKL, Receptor activator of nuclear factor-kappa-B ligand; OPG, Osteoprotegerin; MCP-1, Monocyte chemoattractant protein-1; TNF, Tumor necrosis factor.**

##### Bone tissue

The bone tissue was represented through the main cells and signals that involved in the bone remodeling process. The agents of bone tissue consisted of Osteoclasts, Osteoblasts, and Osteocytes cells, surrounded by an extracellular matrix (ECM) (the non-cell agents). The interactions between these cells were mediated by the receptor activator of nuclear factor-kappa B ligand (RANKL)/RANK/osteoprotegerin (OPG) signaling system. The individual agents of the three types of bone cells were non-mobile agents and each agent occupied one patch in the grid. The RANKL and OPG signals were represented as mobile agents released by osteoblast cells, whilst the osteoclasts were modeled to react as RANK receptors. The osteoclast agents were modeled to perform bone resorption through destroying existing osteocyte agents, after being activated by binding to RANKL. On the other hand, the osteoblast agents were modeled to move toward building new osteocyte agents within the cell-matrix and to control the osteoclast agents’ activation through RANKL/OPG signals. While RANKL was modeled to increase osteoclasts activation, OPG, in turn, is modeled to inhibit this activation by binding to RANKL. In a healthy state, the resorption and formation of new osteocytes are balanced. However, this process is modeled to be altered during the infection by increasing the RANKL concentration and subsequently the osteoclasts activity toward increasing bone destruction. Bone cells were modeled to have the following behavior rules and functions.

###### Osteoclasts

1. During initialization, they are distributed depending on their count in one BMU (Komarova et al., 2003; Paoletti et al., 2012).
2. They go through apoptosis when their ages, which are increased by each time step, reach the lifespan parameter (Manolagas, 2000; Ryser et al., 2009; Lio et al., 2012).
3. New osteoclasts are reproduced every day depending on their reproduction rate parameter.
4. The osteoclasts have RANK receptors on their surfaces (Roodman, 1999), which cause osteoclasts activation through binding to RANKL molecules.
5. Activated osteoclasts move toward destroying neighboring osteocytes cells (Figure 2).

###### Osteoblasts

1. Their initial distribution is based on their count in one BMU (Komarova et al., 2003; Paoletti et al., 2012; Florencio-Silva et al., 2015).
2. When their ages reach their lifespan parameter, they change their status to new osteocytes (Dallas and Bonewald, 2010).
3. New osteoblasts are reproduced every day depending on their reproduction rate parameter.
4. Osteoblasts agents are considered as the source of releasing RANKL. Releasing RANKL is depending on both the reproduction rate parameter and the existence of the bacteria. RANKL modeled to bind to RANK and activate osteoclasts (Figure 2). The releasing process was verified using diffuse instruction in NetLogo.
5. OPG molecules are also released by osteoblasts agents using their reproduction rate and modeled to bind to RANKL in order to inhibit osteoclasts activation.
6. Osteoblasts agents migrate toward forming new osteocytes, after searching for a place where no osteocyte occupies a nearby location to maintain a minimum distance between two osteocytes (Repp et al., 2017).

###### Osteocytes

1. At the initial state, they are distributed in the bone tissue space taking into account a minimum distance between them forming a network of osteocytes cells within the extracellular matrix of bone.
2. Because of their long lifespan that could extend for decades (Franz-Odendaal et al., 2006; Dallas et al., 2013), they were not modeled to undergo reproduction and death phase.
3. Instead, new osteocytes originated from mature osteoblasts and were destroyed by activated osteoclasts.

##### Immune system

It was modeled with three types of innate immune cells namely macrophages, neutrophils, and monocyte-derived macrophages (MDM), which represent the first actors against the bacteria and they respond to the infection in sequential roles (Kaplanski et al., 2003; Strydom and Rankin, 2013; Kim and Luster, 2015). These immune cells were represented as mobile agents “turtles” in the model. The response sequence is modeled on two steps. First, the macrophage and neutrophil agents are modeled to react directly against the bacteria and phagocytose them. In addition, their stimulation is increased by the proliferation of bacteria and regulated by the tumor necrosis factor TNF and transforming growth factor-beta TGF-β cytokines. The second step of defense is modeled to take place if both of macrophages and neutrophils cells could not succeed in eliminating the bacteria by the first 48 h, through recruiting MDM cells to the site of infection. The MDM cells were modeled to be stimulated by the Monocyte chemoattractant protein-1 (MCP-1) cytokine. MDM population is also regulated by the TGF- β signal through a positive feedback loop. TGF-β was represented as mobile agents in the model, while MCP-1 and TNF were represented as variables associated with the infected patches. The functions and rules behind the innate immune cells’ work follow.

###### Macrophages

1. At initialization, macrophages agents are distributed randomly in the model space depending on their initial number, and they are stimulated by the presence of the bacteria.
2. Macrophages go through apoptosis depending on their lifespan parameter, while their ages increase at each time step.
3. New macrophages are reproduced depending on their reproduction rate. The reproduction process follows the uniform distribution function in which the probability of reproducing new macrophages in one time interval is the same for the whole reproduction time.
4. The macrophages agents move around the space and are attracted to the presence of bacteria to phagocytosis them. When one macrophage engulfs a bacterial agent, it dies.
5. The macrophages reproduce TGF-β cytokines that have a double role. They decrease the reproduction of macrophages through a negative feedback loop, while on the second hand, they increase the monocyte production.
6. The macrophages also increase the inflammation by releasing the TNF cytokine as an associated variable that used to interact with neutrophils agents to increase their recruitments.

###### Neutrophils

1. Since they are recruited to the infection site within 4–8 h (Strydom and Rankin, 2013), they were modeled to react against the invader with no delay. Neutrophils agents are created with the initialization of the model and randomly distributed in the modeled area with an initial concentration.
2. Neutrophils go through apoptosis depending on their lifespan parameter, while their ages increase at each time step.
3. New neutrophils agents are produced each day depending on their reproduction rate. Agents’ reproduction is also equally distributed over the time of reproduction.
4. Neutrophils agents follow a random walk toward phagocytosis the bacteria. They die after engulfing the bacteria.
5. They are stimulated by the presence of bacteria and by the pro-inflammatory cytokine TNF that are released by the macrophages. In addition, they are considered as the source of MCP-1, through which the monocytes are recruited.

###### Monocyte-derived-macrophages (MDM)

1. They are recruited to the site 48 h post the bacterial invasion, stimulated by macrophages and neutrophils through MCP-1 and TGF-β cytokines.
2. They have a life cycle that goes through increasing the age and ends by the apoptosis depending on the lifespan parameter (Whitelaw and Bell, 1966; Ginhoux and Jung, 2014; Italiani and Boraschi, 2014).
3. Each day, new MDM agents are modeled to be produced following the uniform distribution function.
4. They are modeled to move toward phagocytosis the bacteria, and they die after engulfing one.
5. They were also considered as a source of TGF-beta signal, through which their count was increased through a positive feedback loop.

The reproduction rate of each of the immune cells was estimated using the method used in the mathematical model of Smith et al. (2011). We assumed that the steady-state *N* is given by *N* = *s/d*, where *s* is the reproduction rate of cells per day and *d* is the clearance rate per day. The clearance rate of macrophages, which is taken from their identified lifespan, is in the range *d* = 7–14 × 10^–2^ day^–1^, implying *s* = 28–115 cell/day, for a steady-state 200–800 cell/mm^3^ (Wallace et al., 1993). In the same way, the reproduction rate for each of neutrophils and monocytes was estimated for steady values taken from an animal model of bone infections (Corrado et al., 2016). The reproduction rate of neutrophils was calculated as 120–700 cell/day for steady-state 250–700 cell/mm3 and *d* = 0.2–1 day^–1^. When the monocytes are activated, they will reproduce at a rate of 4–70 cell/day that estimated for *d* = 0.2–1 day^–1^ and steady-state 20–70 cell/mm^3^.

##### Bacteria

The bacteria in the model were modeled to be affected by several factors; some of them were related to the bacteria themselves, such as their initial inoculum size and reproduction rate. The other factors were those related to the immune cells’ defense and ability to eliminate the bacteria. In their turn, the bacteria dynamics affected the bone tissue health represented by the number of osteocytes or ECM agents. The bacteria were represented in the model as turtles with each individual mapped to one patch, and they propagate depending on their reproduction rate parameter (Table 3). The reproduction rate of bacteria differs in the controlled culture from it in the human body. This rate is difficult to be determined in the human body because of the different factors that have impacts on the bacterial growth in the infected bone such as the location, vascularization, pH, nutrition, and type of prosthesis, if exist. The bacteria characteristics, such as SCV, also have an impact on the growth rate toward decreasing it (Bui et al., 2015). The reproduction rate in the model was identified by the range of 1–24 h to cover several proposed values (Fux et al., 2005; Anwar et al., 2007). The modeled bacterial behaviors rules follow:

**TABLE 3:** The parameters in the model and their values or ranges used.

Parameter	Range in literature	References	Type of study	Range in the model	Step size	Simulation value
Bacteria production-rate	1–24 h	Fux et al., 2005; Anwar et al., 2007	*In vitro*	[1–24] hour	1 h	12 h
Bacteria inoculum size	0–500 CFU/mm^3^	Assumed		[0–500] CFU/mm^2^	10 CFU/mm^2^	5, 50, 500 CFU/mm^2^
Osteocytes initial number	500–900 cell/mm^2^	Vashishth et al., 2000; Goggin et al., 2016	*In vivo*, Human study	1500–2000 cells	–	1790 cells
Osteoblasts production-rate	4 cell/day	Parfitt, 1994; Komarova et al., 2003	*In vivo*, human study	[1–10] cell/day	1 cell/day	4 cell/day
Osteoblasts lifespan	3 months	Manolagas, 2000	Human study	[10–90] day	5 days	50 days
Osteoblasts initial number	800–2000 cells/BMU	Parfitt, 1994; Komarova et al., 2003	*In vivo*, human study	800–2000 cells	–	1000 cells
Osteoclasts production-rate	3 cell/day	Parfitt, 1994; Komarova et al., 2003	*In vivo*, human study	[1–5] cell/day	1 cell/day	3 cell/day
Osteoclasts lifespan	2 weeks	Manolagas, 2000	Human study	[1–14] day	1 day	7 days
Osteoclasts initial number	5–20 cells/BMU	Parfitt, 1994; Komarova et al., 2003	*In vivo*, human study	5–20 cells	–	8 cells
RANKL concentration	10^–6^ mol/cell/day	Ryser et al., 2009	Estimated according to *in vivo* observation	1 μmol/cell/day	1 μmol/cell/day	1 μmol/cell/day
OPG concentration	3.10^–6^ mol/cell/day	Ryser et al., 2009	Estimated according to *in vivo* observation	3 μmol/cell/day	1 μmol/cell/day	3 μmol/cell/day
TGF-β concentration	150–500 pg/ml	Knapp et al., 1998	*In vivo*, human study	1 × 10^–3^ pg/cell/day	–	1 × 10^–3^ pg/cell/day
TNF concentration	0–1000 pg/ml	Corrado et al., 2016	*In vivo*, animal study (murine)	1 × 10^–3^ pg/cell/day	–	1 × 10^–3^ pg/cell/day
MCP-1 concentration	0–2000 pg/ml	Corrado et al., 2016	*In vivo*, animal study (murine)	1 × 10^–3^ pg/cell/day	–	1 × 10^–3^ pg/cell/day
Neutrophil reproduction-rate	—	Estimated		[120–700] cell/hour	50 cells	550 cell/day
Neutrophil lifespan tissue	24–120 h	Rankin, 2010; Bekkering, 2013	*In vivo*, human study, mice study	[24–120] hour	6 h	60 h
Monocyte lifespan	24–120 h	Whitelaw and Bell, 1966; Ginhoux and Jung, 2014; Italiani and Boraschi, 2014; Patel et al., 2017	*In vivo*, human study, mice study	[24–120] hour	5 h	60 h
Monocyte reproduction rate	–	Estimated		[4–70] cell/day	50 cell/day	150 cell/day
Macrophage lifespan	1–14 days	Parwaresch and Wacker, 1984	*In vivo*, rat study	[24–300] hours	6 h	24 h
Macrophage reproduction-rate	–	Estimated		[28–115] cell/day	10 cell/day	550 cell/day

**BMU, Basic Multicellular Unit.**

1. Bacteria agents are randomly distributed depending on their initial inoculum size.
2. Their life cycle goes through the reproduction phase depending on their reproduction rate parameter.
3. The bacteria spread and move toward invading the bone tissue.
4. The bacteria go through death rate which symbolizes the run out of their resources of survival.
5. The existence of bacteria stimulates the immune cells to react against the invasion starting by macrophages and neutrophils agents.

#### Process Overview and Events Sequence

The simulation was launched using a function that mainly sets all the variables to their initial values, in addition to carrying out the following tasks. First, it established a basic grid of patches (151 × 101 patches). Second, it initiated the bone cells patches according to their percentage of bone cells count and allocated them randomly. Third, it created numbers of macrophage and neutrophil agents corresponding to their initial value and distributed them randomly. Fourth, it created a number of bacteria agents (turtles) respecting their chosen initial value, and randomly allocated each cell to one patch on the adjacent part of the grid. Finally, it created numbers of signaling agents (TGF-β, RANKL, OPG) corresponding to their initial concentration level, and distributed them randomly.

After initialization, the simulation started looking through the set of functions and rules over time and were repeated up to the end of simulation either by eliminating the bacteria or reaching the determined simulation time (300 h). After the initialization of bacteria, proliferation was modeled using binary fission depending on varied reproduction time. The fission function was modeled to follow the normal distribution over the time of reproduction (Table 3). For one bacteria to undergo fission, it must search for one empty neighboring patch of the bone tissue; otherwise, no fission would take place. At each time step, the agents updated their location and moved upon their functions and rules, the bacteria moved toward invading the bone tissue, the immune cells moved toward engulfing the bacteria, and the osteoclasts and osteoblasts updated their location toward resorbing and forming new osteocytes.

The population and distribution of each type of agent were calculated at each time step, if some agents reached their lifespan, they went under apoptosis, otherwise, they increased their age. At the time of reproduction, new agents were created upon their reproduction rate and allocation conditions. During their life, each type of agents performed its rules and functions that described in section Agent Types, Rules, and Parameters.

During the simulation and at each time step, the populations of a pre-defined set of agents were saved to “.csv” file. These output files were identified by the initial conditions of the simulation. Multiple runs of the same simulation were saved to the same output file in order to analyze the model outcomes for the same initial conditions. The progress of the infection over time and space during the simulation is shown in the simulator interface (Figure 1). Changes in the agents’ counts were monitored through time graphs for each type of agent in the NetLogo interface.

### Simulated Experiments

The goals of the simulation were to illustrate the relationships between variation in the bone cell population and the evolution of infectious processes. The goal was also to investigate the efficiency of the innate immune system in defending the bacteria during the first stage of the BJI for several scenarios without treatment intervention. Further, the simulations were conducted to detect sensitive parameters that affected the dynamics of the system.

To verify the model stability and performance, and to investigate the effect of bacterial inoculum size on the system dynamics, the initial number of bacteria were adjusted to three different values (5, 5 × 10, 5 × 10^2^ CFU/mm^2^). These values were assumed to represent three different infected inoculum states – low (5 CFU/mm^2^), medium (5 × 10 CFU/mm^2^) and high (5 × 10^2^ CFU/mm^2^). We ran the simulations for *n* = 100 iterations for each of the infected inoculum state, while all other changeable parameters were set to the median of their range (Table 3). For each iteration, the dynamics of the population for bacteria, osteocytes, and neutrophils agents for a time duration of 300 h (*t* = 300 ticks) was tracked. The generated data at each time click (1 h) were saved to an output data (.csv) file for analysis. Subsequently, the mean and standard deviation for the population at each time step for each type of these agents was quantified. Further, the relation between two agents over time under the same initial conditions was analyzed. We benefitted from the 3D surface graphs and the generated data from the previous step to analyze the relationships between each of bacteria and neutrophils populations over time, bacteria and osteocytes populations over time, and neutrophils and osteocytes populations over time.

## Results

### Characterizing the Agent Interactions and Parameters Values

The systematic search process identified 42 articles for bone cell characterizations, 48 articles for immune cell characterization, 12 articles for bacteria characterization, and 29 articles for interactions (Supplementary Figures S1–S4). Following the systematic literature search, the agent interactions and the parameter ranges were identified from the retrieved articles. Flowcharts of the review steps along with the list of all articles used for the study are reported in Supplementary Figures S1–S4 and Supplementary Table S2.

### The Developed Agent-Based Model of BJI

A representative example of the model space was taken during one simulation at three different times (Figure 1). The initial state of the model was represented at the beginning of the simulation (0 h), for initial inoculum infection state of (5 × 10^2^ CFU/mm^2^), where this initial number of bacteria were randomly distributed in the adjacent surface. It also demonstrated the low presence of immune cells macrophages and neutrophils, and the initial state of bone tissue before being damaged (Figure 1A). The progress of the infection during the simulation (60 h) later showed that the bacteria propagated toward entering the bone tissue and destroying it. The monocytes also had been activated in this step besides the other immune cells (Figure 1B). The simulation state at (150 h) showed the system after 6 days, where the damage happened to the bone tissue, and the bacteria count decreased because of engulfing by the immune cells (Figure 1C). This model is 2D what makes it suitable to represent trabeculae bone, but it could be also considered as a cross-section of cortical bone.

### Simulated Experiments Output

**FIGURE 3:**
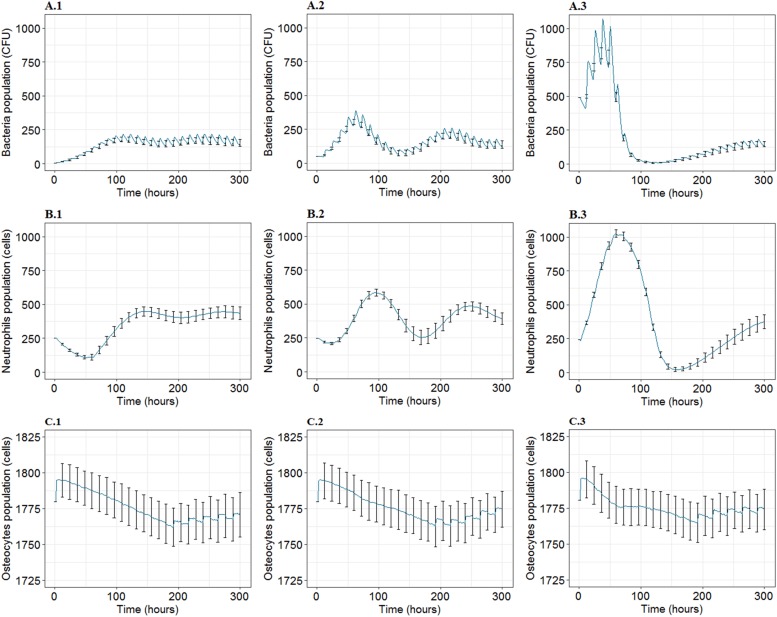
The mean and standard deviation (SD) for 100 iterations for agent populations over time, *t* = 300 h, at three inoculum infection states of bacteria (5, 50, 500 CFU/mm^2^). **(A1–A3)** Show the mean and SD of the bacteria population over time. **(B1–B3)** Show the mean and SD corresponding to neutrophils population. **(C1–C3)** Represent mean and SD for osteocytes population. First, second and third columns represent the results for inoculum infection states of low (5 CFU/mm^2^), medium (5 × 10 CFU/mm^2^), and high (5 × 10^2^CFU/mm^2^) bacteria respectively.

The mean and the standard deviation of the dynamics of the population for 100 iterations under different infection inoculum states (low, medium, high) revealed interesting behavior for each of the bacterial, neutrophil (PMN), and osteocyte population (Figure 3). The bacterial population inclined toward the same steady non-null counts, regardless of the inoculum state (Figures 3A1–A3). In addition, the population intensity of the first few days was proportional to inoculum size. It was also observed that the behavior of the bacteria varied compared to the mean behavior with small variance magnitude even if the output illustrated (by the effect of time characteristic) a high-frequency oscillation in the bacteria population. At the same time, it showed low fluctuation in the context of the general trend of the bacterial population (Figures 3A1–A3). It was also observed that the bacterial population faced quasi-extinction, followed by re-growth for the inoculum in the medium and high infection state for all 100 iterations.

On the other hand, the PMN dynamics followed the evolution of the bacteria with a slight delay, reaching a non-zero stable level on the 12th day. In addition, it illustrated that the PMN population was not subject to rapid variations represented by the low-frequency oscillations, which are comparatively smoother than those introduced by the bacteria (Figures 3B1–B3). Asymptotic behavior of PMN population for the three inoculum states proposed a non-null mean value with fixed frequency oscillations and decreasing magnitude with time (Figures 3B1–B3).

Concerning the bone tissue loss in each state, the osteocyte population dynamics was similar in mean population intensity, while it differed from the mean behavior with important variance magnitudes whatever the inoculum. The bone cell population predicted a trend of unexpected high-frequency oscillations after *t* = 200 h (Figures 3C1–C3).

Comparisons of system response for different inoculum states and over time for the same type of agent population suggested that the high inoculum infection state was associated with close to elimination of bacteria population and full elimination of PMN population. It was also associated with the highest population counts for both agent types in the first few days of the infection (Figure 3). The results presented that bacteria and PMN tended toward a steady asymptotic non-null state, regardless of the inoculum infection state. With regards to bone tissue damage, the first response stage had a similar degradation phase of the bone infected site represented by decreasing count of osteocytes for each inoculum state, where it was noted that osteocytes passed by a common minimum level by the 7th-8th day for all three inoculum states, representing 2% of the loss in the infected site mass (Figure 3). During the recovery phase, it was observed that the smallest inoculum infection state caused the most severe and relatively stable loss on bone cells by 12th day, the medium inoculum infection state showed better progressive recovery, and the high inoculum infection state displayed an intermediate recovery, with sub-optimum recovery at 300 h. The displayed loss of bone tissue compared to baseline for each inoculum infection state was 1.4, 1.2, and 1%, respectively (Figure 3).

**FIGURE 4:**
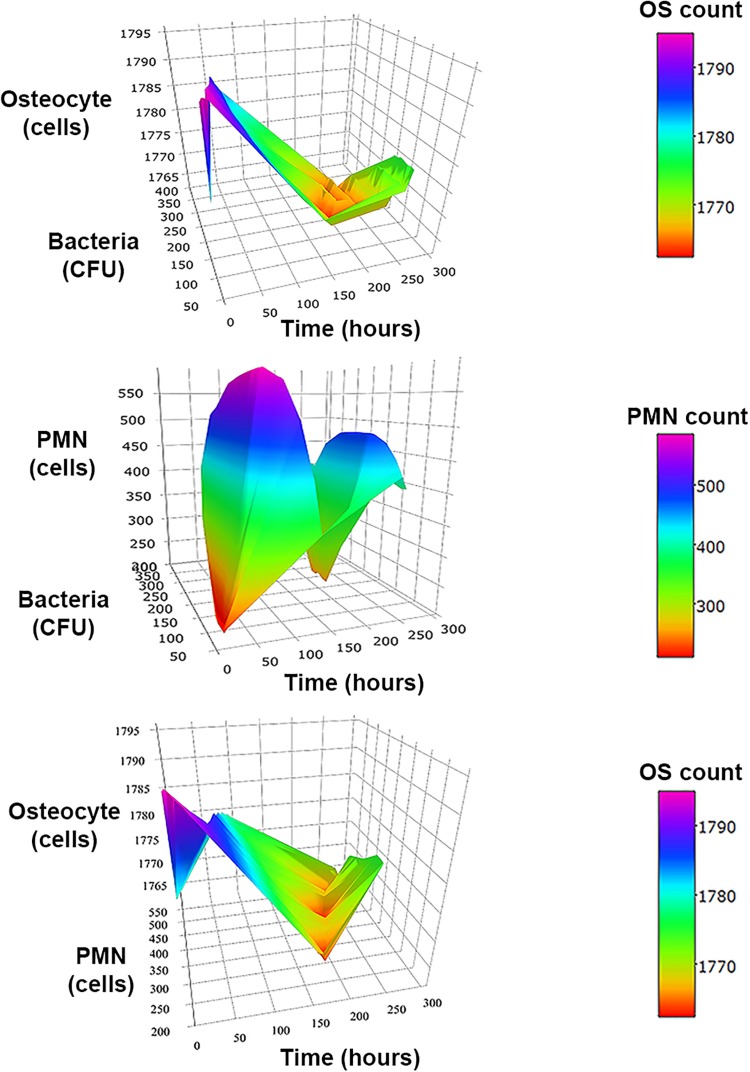
3D surface graphs to analyze the relationships between two types of agents over time at inoculum infection states of bacteria (50 CFU/mm^2^). In the top, the graph shows the relationship between bacteria vs. osteocytes (OS) population over time for the three initial inoculum values. The second row corresponds to the osteocytes (OS) vs. neutrophils (PMN) population over time. The third row shows the relationship of neutrophils (PMN) vs. bacteria populations over time.

The 3D representation of the relation between two types of agents over time suggested minimum levels of bacteria and PMN population count around the fourth day of infection, while it suggested a delay in minimum levels of population in osteocytes count with regard to the minimum levels of bacteria and PMN (Figure 4).

## Discussion

The incidence rate of post-operative/post-traumatic infections is showing an important annual increase due to the rising in arthroplasties procedures associated with mounting risk factors such as diabetes and peripheral vascular disease, in addition to the variation in the population’s age structure (Walter et al., 2012). BJI are complex and characterized by different patterns of bacteria progression and different hypotheses in the literature. The lack of strong evidence is exacerbated by indefinite possible dynamic processes and interactions at cellular and molecular levels. This infection is characterized by high divergence during inter and intra infected cases, and by multi pre-defined variables, making it difficult to rely on classical statistical models. By integrating the ABM modeling technique along with the knowledge from the literature review, this study introduced an innovative *in silico* experimental environment to explain the hypotheses and knowledge in the literature and investigate the role and dynamics of other interactions that could be proposed. The developed model offers a means to test the impact of several factors on the infected tissue. Through this model, this study illustrated the role of bacterial virulence and host defense state that identify the consequences of bacterial invasion to the bone. Although the simulations are at the preliminary levels with regards to their accuracy and validity, several interesting outcomes were observed that would certainly provide insights into the BJI dynamics.

### Observation

For the proposed inoculum infection states, on the12th day, the observed bacterial population for the inoculum of the order of 100 was stable and not extinct, which could also be considered as an indication for latent infection. Since the PMN populations followed, in the same manner, the bacteria populations but in a slight delay to reach the stable non-null levels on the 12th day, it could also be used as an indication of the latent infection. If so, it should be validated in terms of PMN count in the biological laboratory test. In addition, the relationship between the osteocytes and the inoculum infection state raises the question of whether it states *a restitutio ad integrum* for the medium infection state that introduced the best progressive recovery.

Further, the observed stable non-extinct level of bacteria population 10 days after the infection showed the inability of the innate immune cells to completely eliminate the bacteria. This points out the imperative role of further defense methods: the adaptive immune response, which initiated by the 4th–7th day post the infection, and the need for therapeutic intervention.

In terms of population fluctuations (intra-simulation), the observed small variance within the bacterial population dynamics highlighted their stable behavior even with the rapid, strong oscillation which could be an artifact of the internal clock management in NetLogo algorithm. The fluctuations observed in the PMN population had a less important effect compared to the bacterial state, which reflects a spatial impact. In fact, greater the probability that a PMN encountered a bacterium, more likely it would have reacted, which ultimately reduced the fluctuations.

On the other hand, the predicted high-frequency oscillating trend of the osteocytes population after *t* = 200 h was unexpected. It was expected to display a rapid decreasing phase, and then a slower increasing phase. This considerable fluctuations in the osteocytes population appeared interesting as it could partially explain the inter-individual variability. Further, these fluctuations increased in intensity over time, raising the question if it could be explained as a pre-chaotic behavior “positive feedback.” It was also noted that the PMN and the bacteria populations reach their minimum levels simultaneously before rising again. This weakness in the innate immune response could provide a better possible window to start the therapeutic treatment.

### Strengths

The BJI system model developed in this study is the first to incorporate experimental data with the ABM modeling approach and extract information in the form of a dynamic system and it lays the foundations for an in-depth and detailed BJI simulation system. The interactions between the agents and signals provide a comprehensive ability to analyze the system considering the spatial characteristics and the inter-agent variability. Additionally, the observed results corroborate with clinically and microbiologically available pathophysiological pathways and they are generally in line with expectations, as that the investigated populations move toward equilibrium (Wagner et al., 2003). This model has also shown its predictive ability for the evolution of bone mass with respect to bacterial inoculum states and time. The proposed model framework introduces a flexible and interactive virtual laboratory to test and explain several existing hypotheses or even to design new ones.

### Limitations

One of the major limitations of the ABM approach is that it generally cannot be released simply, unless the entire model is given. Evidence is also difficult to obtain, and implementation details can cause many problems. For that, we had to start with a less complex model (at the same time integral one) and to build later upon it. The developed model presents certain limitations, and all the results should be evaluated within the framework of these limitations. The first limitation is model validation due to the lack of experimental data, which might be acquired at a later stage (e.g., the bone mass). The 2D representation represents a limitation with regards to the ability of agents to interact in a 3D space. However, we adopted the simplicity in building this first model aiming at developing a feasible modeling framework and understanding the complex integration of available physiological data with the ABM modeling framework. Future work is aimed at enhancing the model by a more realistic architecture, tissue specification, and patient-based data. The current model lacks the second stage of the immune response, the adaptive immune response, which is necessary to investigate the further progression of the infection. The bacteria biofilms, which are lacking in the current model, also play an important role in identifying the behavior of the bacteria itself and the system response due to their resistance to immune defense and antibiotic agents. Since biofilms are common during this infection, it is worthwhile to model them in the future. In this model we were limited to *S. aureus*, other pathogens will be investigated in the future. In this first model, the BJI was modeled without prostheses, which will be added in the future. Other agents including bacterial survival factors have a significant contribution in introducing an additional approximation to mimic the real system. The adaptability of the present model depends on the type of modifications that are foreseen. All modifications needed would be investigated through the same process we described in this study: first, identify if (i) new agents (e.g., cells) should be integrated and (ii) already modeled interactions are sufficient or should be modified or completed by new ones; second, list all relevant parameters and parameters values related to these modifications through literature search; finally, model agents, messengers and implement parameters values in the new model. This process can be applied if we want to introduce variants or new pathogens that may interact with each other and with the existing agents or if we want to model cancellous bone rather than cortical bone or if children and adults BJIs differ from critical aspects.

### Vision

This work has drawn attention toward the incorporation between modeling power and current literature using the ABM modeling framework of infection dynamics. It also highlights the progression of the simulation environment that generates data that would be used in the extraction and synthesis of the model in the form of differential equation systems. This model could in fine be integrated within a global predictive approach to propose more personalized therapeutic treatments. The future global approach would combine microbiological and imaging data of the patient to extract infection parameters (bacteria, immune system) and patient-specific bone morphology. As further data are made available, the model would be refined to better estimate an in-depth comprehension of BJI’s pathophysiology. Furthermore, studying the dynamics of the BJI including critical parameters such as the agent interactions and cross-talk signals could make it possible to test new or alternative therapeutic pathways for their efficacy.

## Author Contributions

SAl drafted the manuscript and was responsible for applying the computational algorithm, implementing the ABM model, doing the simulations, and processing the results. TL, SAn, and ES were responsible for revising the manuscript, for the design and interpretation of the simulation, and ABM results. TL, VL, SAn, and ES supervised the work.

## Conflict of Interest

SAl and VL were employed by company Tekliko SARL. The remaining authors declare that the research was conducted in the absence of any commercial or financial relationships that could be construed as a potential conflict of interest.
