# Supplementary material for: Modeling Early Stages of Bone and Joint Infections Dynamics in Humans: A Multi-Agent, Multi-System Based Model
Source: Front Mol Biosci. 2020 Mar 12;7:26. doi: 10.3389/fmolb.2020.00026 (PMC7080862; doi:10.3389/fmolb.2020.00026)
Supplement: Supplementary file 1 [file Data_Sheet_1.PDF]

Supplementary Material

Modeling early stages of bone and joint infections dynamics in humans: a multi-agents, multi-systems based model

Salma Alsassa[1,2*], Thomas Lefèvre[3,4], Vincent Laugier[2], Séverine Ansart[1,5], Eric Stindel[1,5]

*** Correspondence:**
Salma Alsassa
engsalma80@gmail.com

1 Literature review for agents interactions and parameters values identification

1.1 Method

The first step toward building our ABM model of BJI is describing this system and its behaviors in general and characterizing its components, their rules, and the interactions between them on different scales. We based on a literature review to retrieve agents' behaviors, rules and interactions, relevant parameters and parameters ranges of acceptable values. By giving some parameters a range of values, we enrich our model with the capacity of simulating different behaviors of the agents and testing several hypotheses.

Relying on PubMed and ScienceDirect databases, we explore the related journal paper works from their inception to date, July 2017. In order to do that, we identified the research questions, the including and the excluding criteria for each key of research. We took benefits of quantitative description in the related computational and mathematical model beside the in-vivo or in-vitro experiments. The queried terms are classified into four groups, the tissue cellular system, the innate immune system, the bacteria system, and the interactions between the components of these systems. In the following, we will itemize each group of terms with both including and excluding criteria.

I. For characterizing the bone cells, their lifespan, their main function 'bone remodeling' and the signals that involving in, we proposed the following questions and criteria:

a) The search questions were:
   1. Osteoblast*AND (half-life OR lifespan OR life-span OR (reproduc* rate))
   2. Osteoclast*AND (half-life OR lifespan OR life-span OR (reproduc* rate))
   3. Osteocyte* AND (half-life OR lifespan OR life-span OR (reproduc* rate) OR ratio OR density)
   4. Bone remodeling process AND (signal* OR RANK OR RANKL OR OPG OR osteoprotegerin OR receptor activator of nuclear factor kappa*)

b) The inclusion criteria were human studies, articles on bone formation and bone resorption, quantitative studies and bone biology.

c) The exclusion criteria were excluding the article related to specific cases and diseases, the article related to genetic researches, pathway mediated, or some treatment effects studies.

II. In order to identify the immune cells, quantify their lifespan, define the role of TGF signal on the immune response, and characterize the behavior of innate immune response, we proposed the following questions and criteria:

a) The search questions were:

1. Neutrophil* AND (half-life OR lifespan OR life-span OR (reproduc* rate))
2. Monocyte* AND (half-life OR lifespan OR life-span OR (reproduc* rate))
3. (macrophage* OR (resident macrophage*))AND (half-life OR lifespan OR life-span OR (reproduc* rate))
4. (innate immun* OR innate immun* system) AND ((math* OR computation*) model) AND (macrophage OR monocyte OR neutrophil)
5. (tgf* OR Transforming growth factor) AND (regule* OR interact* OR signal*) AND (innate immunity OR innate immune system OR innate immune cells AND (macrophage* OR neutrophil* OR monocyte*))

b) The inclusion criteria were: human studies, articles handling lifespan and regulation leukocytes, and innate immune response biology.

c) The exclusion criteria were: excluding the article related to specific cases and diseases,the article related to labeling methods, receptors patterns or gene expression studies.

III. In order to characterize the bacteria agents, their growth and population in the site of infection, we proposed the following questions and criteria:

a) The search questions were:

1. (*Staphylococcus aureus* AND bacteria) AND (count OR population) AND (osteomyelitis OR bone infection) AND animal model
2. *Staphylococcus aureus* AND (reproduc* rate OR generat* time OR growth rate) AND (osteomyelitis OR bone infection)
3. (computation* OR math*) AND model AND (osteomyelitis OR bone infection)

b) The inclusion criteria were *Staphylococcus aureus* population or rate, animal model of BJI for (1, 2).

c) The exclusion criteria were implant material, in vitro studies, children osteomyelitis, antibodies studies, or image studies.

IV. To investigate the interactions between the agents of the three previous systems, we proposed the following questions:

a) The search questions were:

1. (Macrophage* OR neutrophil* OR monocyte*) AND (bacteria OR *Staphylococcus aureus*) AND (bone infection* OR osteomyelitis)
2. (Osteoclast* OR osteoblast* OR osteocyte*) AND (bacteria OR *Staphylococcus aureus*) AND (bone infection* OR osteomyelitis)
3. (RANK OR RANKL OR OPG OR osteoprotegerin OR receptor activator of nuclear factor kappa* OR TGF OR transforming growth factor) AND (bacteria OR *Staphylococcus aureus*) AND (bone infection* OR osteomyelitis)
4. (macrophage* OR monocyte* OR neutrophil*) AND (osteoclast* OR osteoblast* OR osteocyte*) AND (osteomyelitis OR bone infection)

b) The inclusion criteria were *Staphylococcus aureus* or osteomyelitis studies, animal model or human studies, signals and regulations studies, matching keywords articles.

c) The exclusion criteria were implant material, children osteomyelitis, antibodies, and gene expression or image studies.

2.1 Results

The scoping review method yielded a set of papers for each research question. In the fig. S1-S4, the flowchart shows the resulting number of paper for each review step, and in Table. S2 the resulting paper.

2 Supplementary Figures

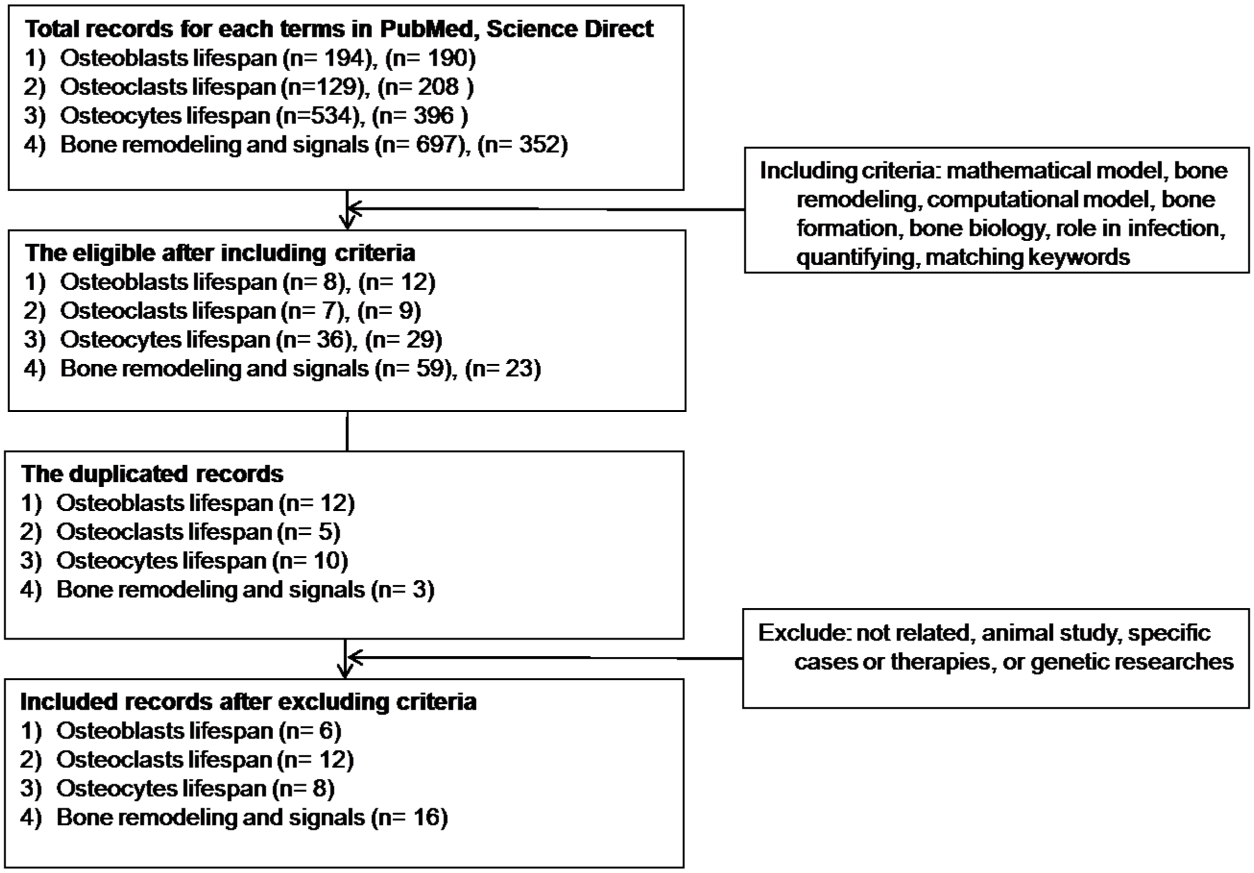

Fig. S1. Flowchart of the review method for the bone cells.

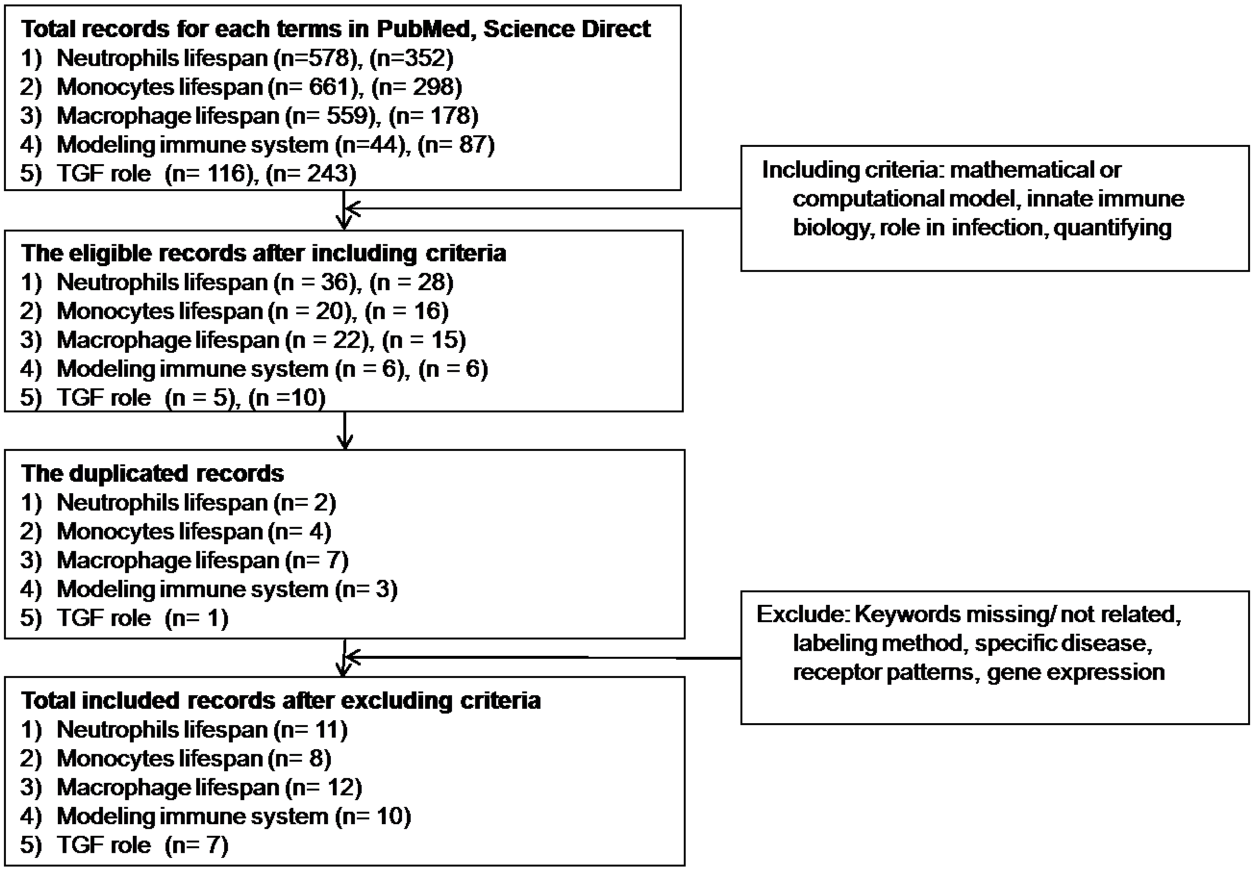

Fig. S2. Flowchart of the review method for the immune system.

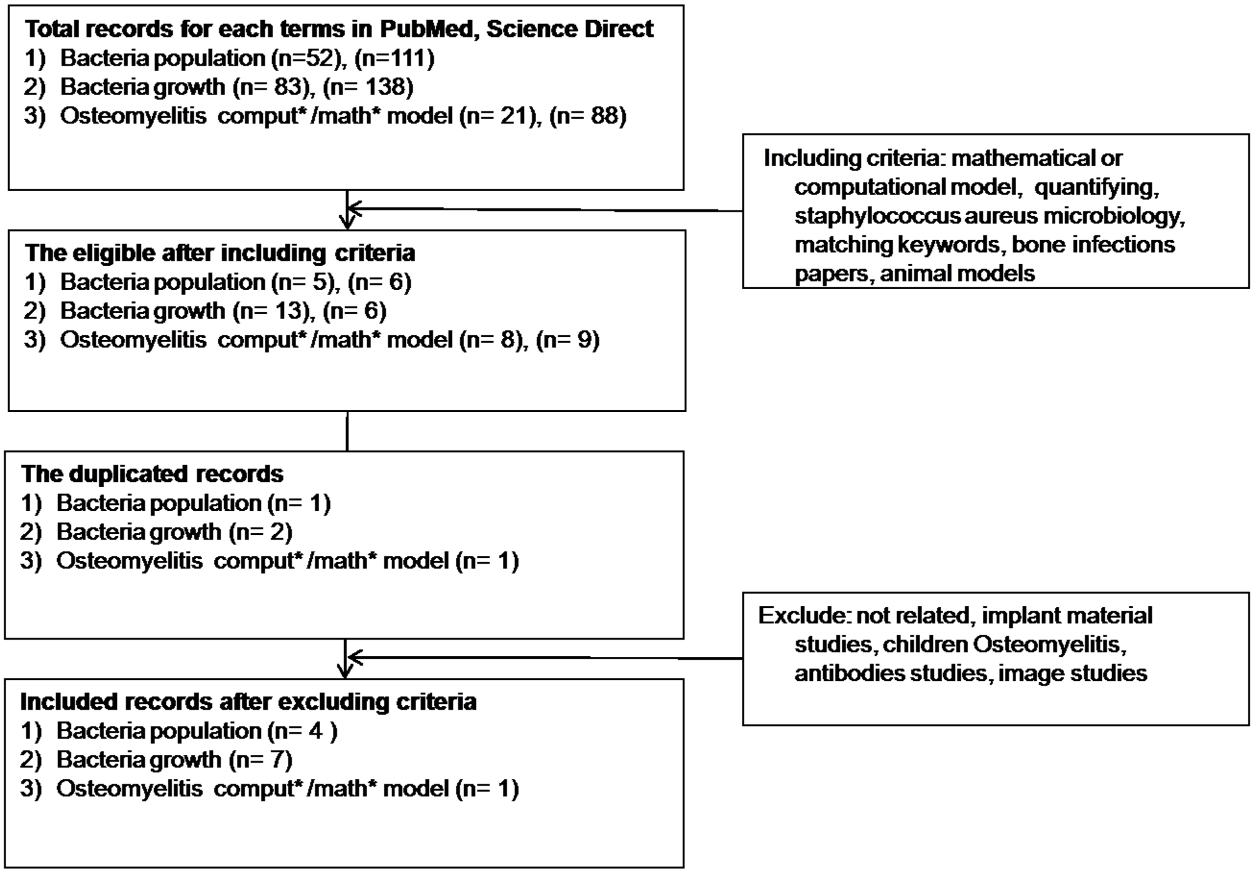

Fig. S3. Flowchart of the review method for the infectious system.

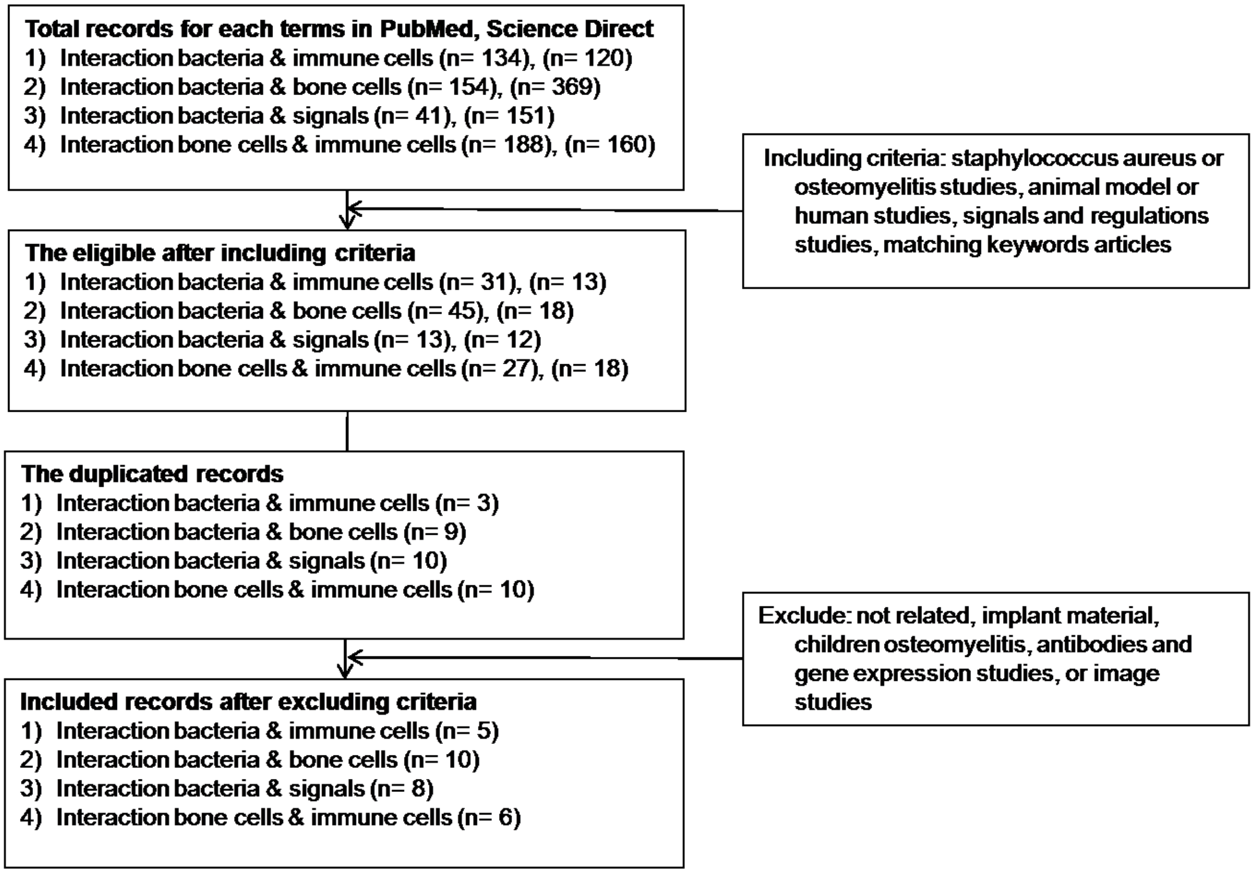

Fig.S4. Flowchart of the review method of the interactions between agents.

3 Supplementary Tables

Table. S1. Table of research questions used in the bibliography, including and excluding criteria

Search Equations	Including Criteria	Excluding Criteria
**Bone cells**		
1. Osteoblast*AND (half-life OR lifespan OR life-span OR (reproduc* rate))<br>2. Osteoclast*AND (half-life OR lifespan OR life-span OR (reproduc* rate))<br>3. Osteocyte* AND (half-life OR lifespan OR life-span OR (reproduc* rate) OR ratio OR density)<br>4. Bone remodeling process AND (signal* OR RANK OR RANKL OR OPG OR osteoprotegerin OR receptor activator of nuclear factor kappa*)	human studies, articles on bone formation and bone resorption, quantitative studies, bone biology	the article related to specific cases and diseases, the article related to genetic researches, pathway mediated, or some treatment effects studies
**Innate Immune Cells**		
1. Neutrophil* AND (half-life OR lifespan OR life-span OR (reproduc* rate))<br>2. Monocyte* AND (half-life OR lifespan OR life-span OR (reproduc* rate))<br>3. (macrophage* OR (resident macrophage*)) AND (half-life OR lifespan OR life-span OR (reproduc* rate))<br>4. (innate immun* OR innate immun* system) AND ((math* OR computation*) model) AND (macrophage OR monocyte OR neutrophil)<br>*5.* (tgf* OR Transforming growth factor) AND (regule* OR interact* OR signal*) AND (innate immunity OR innate immune system OR innate immune cells AND (macrophage* OR neutrophil* OR monocyte*))	human studies, articles handling lifespan and regulation leukocytes, and innate immune response biology	excluding the article related to specific cases and diseases, the article related to labeling methods, receptors patterns or gene expression studies
**Bacteria**		
1. (*Staphylococcus aureus* AND bacteria) AND (count OR population) AND (osteomyelitis OR bone infection) AND animal model<br>2. *Staphylococcus aureus* AND (reproduc* rate OR generat* time OR growth rate) AND (osteomyelitis OR bone infection)<br>3. (computation* OR math*) AND model AND (osteomyelitis OR bone infection)	*Staphylococcus aureus* population or rate, animal model of BJI for (1, 2)	implant material, in vitro studies, children osteomyelitis, antibodies studies, or image studies
**Interactions**		
1. (Macrophage* OR neutrophil* OR monocyte*) AND (bacteria OR *Staphylococcus aureus*) AND (bone infection* OR osteomyelitis)<br>2. (Osteoclast* OR osteoblast* OR osteocyte*) AND (bacteria OR *Staphylococcus aureus*) AND (bone infection* OR osteomyelitis)<br>3. (RANK OR RANKL OR OPG OR osteoprotegerin OR receptor activator of nuclear factor kappa* OR TGF OR transforming growth factor) AND (bacteria OR *Staphylococcus aureus*) AND (bone infection* OR osteomyelitis)<br>4. (macrophage* OR monocyte* OR neutrophil*) AND (osteoclast* OR osteoblast* OR osteocyte*) AND (osteomyelitis OR bone infection)	*Staphylococcus aureus* or osteomyelitis studies, animal model or human studies, signals and regulations studies, matching keywords articles	implant material, children osteomyelitis, antibodies and gene expression studies, or image studies

Table. S2. Table of the resulting articles of the literature review.

Osteocyte lifespan

Alliston, T. “Biological Regulation of Bone Quality.” *Current osteoporosis reports* 12.3 (2014): 366–375. Web.

Buenzli, P. R. “Osteocytes as a Record of Bone Formation Dynamics: A Mathematical Model of Osteocyte Generation in Bone Matrix.” *Journal of theoretical biology* 364.Journal Article (2015): 418–427. Web.

Buenzli, P. R., and N. A. Sims. “Quantifying the Osteocyte Network in the Human Skeleton.” *Bone*75.Journal Article (2015): 144–150. Web.

Capulli, Mattia, Riccardo Paone, and Nadia Rucci. “Osteoblast and Osteocyte: Games without Frontiers.” *Bone: A dynamic and integrating tissue* 561.Journal Article (2014): 3–12. Web.

Florencio-Silva, Rinaldo et al. “Biology of Bone Tissue: Structure, Function, and Factors That Influence Bone Cells.” *BioMed Research International* 2015 (2015): n. pag. *PubMed Central*. Web. 14 Aug. 2017.

Prideaux, M., D. M. Findlay, and G. J. Atkins. “Osteocytes: The Master Cells in Bone Remodelling.” *Current opinion in pharmacology*28.Journal Article (2016): 24–30. Web.

Qiu, S. et al. “Relationships between Osteocyte Density and Bone Formation Rate in Human Cancellous Bone.” *Bone* 31.6 (2002): 709–711. Print.

Teti, Anna, and Alberta Zallone. “Do Osteocytes Contribute to Bone Mineral Homeostasis? Osteocytic Osteolysis Revisited.” *Bone* 44.1 (2009): 11–16. *ScienceDirect*. Web.

Osteoclast lifespan

Bar-Shavit, Zvi. “The Osteoclast: A Multinucleated, Hematopoietic-Origin, Bone-Resorbing Osteoimmune Cell.” *Journal of Cellular Biochemistry* 102.5 (2007): 1130–1139. *Wiley Online Library*. Web.

Buenzli, P. R., P. Pivonka, and D. W. Smith. “Spatio-Temporal Structure of Cell Distribution in Cortical Bone Multicellular Units: A Mathematical Model.” *Bone* 48.4 (2011): 918–926. *ScienceDirect*. Web.

Chambers, T.j. “The Birth of the Osteoclast.” *Annals of the New York Academy of Sciences* 1192.1 (2010): 19–26. *Wiley Online Library*. Web.

Charles, Julia F., and Antonios O. Aliprantis. “Osteoclasts: More than ‘Bone Eaters.’” *Trends in Molecular Medicine* 20.8 (2014): 449–459. *ScienceDirect*. Web.

Del Fattore, A., A. Teti, and N. Rucci. “Osteoclast Receptors and Signaling.” *Archives of Biochemistry and Biophysics* 473.2 (2008): 147–160. Web.

Lemaire, Vincent et al. “Modeling the Interactions between Osteoblast and Osteoclast Activities in Bone Remodeling.” *Journal of Theoretical Biology* 229.3 (2004): 293–309. *ScienceDirect*. Web.

Mellis, D. J. et al. “The Skeleton: A Multi-Functional Complex Organ: The Role of Key Signalling Pathways in Osteoclast Differentiation and in Bone Resorption.” *The Journal of endocrinology* 211.2 (2011): 131–143. Web.

Roodman, G. David. “Cell Biology of the Osteoclast.” *Experimental hematology* 27.8 (1999): 1229–1241. Web.

Shead, Elizabeth F. et al. “Osteoclast Function, Bone Turnover and Inflammatory Cytokines during Infective Exacerbations of Cystic Fibrosis.” *Journal of Cystic Fibrosis* 9.2 (2010): 93–98. *www.cysticfibrosisjournal.com*. Web.

Soysa, NiroshaniSurangika, and Neil Alles. “Osteoclast Function and Bone-Resorbing Activity: An Overview.” *Biochemical and biophysical research communications* 476.3 (2016): 115–120. Web.

Tanaka, S. et al. “Molecular Mechanism of the Life and Death of the Osteoclast.” *Annals of the New York Academy of Sciences* 1068.Journal Article (2006): 180–186. Print.

Wu, X. et al. “Osteoclast Apoptosis: The Role of Fas in Vivo and in Vitro.” *Endocrinology* 144.12 (2003): 5545–5555. Web.

Osteoblast lifespan

Franchimont, N., S. Rydziel, and E. Canalis. “Transforming Growth Factor-β Increases Interleukin-6 Transcripts in Osteoblasts.” *Bone* 26.3 (2000): 249–253. Web.

Jilka, R. L. et al. “Quantifying Osteoblast and Osteocyte Apoptosis: Challenges and Rewards.” *Journal of bone and mineral research : the official*

journal of the American Society for Bone and Mineral Research 22.10 (2007): 1492–1501. Web.

Kollet, O. et al. "Regulatory Cross Talks of Bone Cells, Hematopoietic Stem Cells and the Nervous System Maintain Hematopoiesis." *Inflammation & allergy drug targets* 11.3 (2012): 170–180. Print.

Komarova, Svetlana V. et al. "Mathematical Model Predicts a Critical Role for Osteoclast Autocrine Regulation in the Control of Bone Remodeling." *Bone* 33.2 (2003): 206–215. *ScienceDirect*. Web.

Manolagas, Stavros C. "Birth and Death of Bone Cells: Basic Regulatory Mechanisms and Implications for the Pathogenesis and Treatment of Osteoporosis." *Endocrine Reviews* 21.2 (2000): 115–137. *academic.oup.com*. Web.

Ryser, Marc D, Nilima Nigam, and Svetlana V Komarova. "Mathematical Modeling of Spatio-Temporal Dynamics of a Single Bone Multicellular Unit." *Journal of Bone and Mineral Research* 24.5 (2009): 860–870. *Wiley Online Library*. Web.

Bone Remodeling and signals

Anandarajah, Allen P. "Role of RANKL in Bone Diseases." *Trends in Endocrinology & Metabolism* 20.2 (2009): 88–94. Web.

Bahar, H. et al. "Molecular Signaling in Bone Regeneration." *Critical reviews in eukaryotic gene expression* 17.2 (2007): 87–101. Print.

Boyce, Brendan F., and Lianping Xing. "Functions of RANKL/RANK/OPG in Bone Modeling and Remodeling." *Archives of Biochemistry and Biophysics* 473.2 (2008): 139–146. *ScienceDirect*. Web. Highlight Issue: Bone Remodeling: Facts and Perspectives.

Henriksen, Kim et al. "Local Communication on and within Bone Controls Bone Remodeling." *Bone* 44.6 (2009): 1026–1033. Web.

Iolascon, G., G. Resmini, and U. Tarantino. "Inhibition of RANK Ligand: A New Option for Preventing Fragility Fractures." *Aging clinical and experimental research* 23.2 Suppl (2011): 28–29. Print.

Kardas, D., U. Nackenhorst, and D. Balzani. "Computational Model for the Cell-Mechanical Response of the Osteocyte Cytoskeleton Based on Self-Stabilizing Tensegrity Structures." *Biomechanics and modeling in mechanobiology* 12.1 (2013): 167–183. Web.

Kiechl, Stefan et al. "The Osteoprotegerin/RANK/RANKL System: A Bone Key to Vascular Disease." *Expert Review of Cardiovascular Therapy* 4.6 (2006): 801–811. *Taylor and Francis+NEJM*. Web.

Nakashima, T. "Coupling and Communication between Bone Cells." *Clinical calcium* 24.6 (2014): 853–861. Print.

Paoletti, N. et al. "Multilevel Computational Modeling and Quantitative Analysis of Bone Remodeling." *IEEE/ACM Transactions on Computational Biology and Bioinformatics* 9.5 (2012): 1366–1378. *IEEE Xplore*. Web.

Pivonka, Peter et al. "Model Structure and Control of Bone Remodeling: A Theoretical Study." *Bone* 43.2 (2008): 249–263. *ScienceDirect*. Web.

---. "Theoretical Investigation of the Role of the RANK–RANKL–OPG System in Bone Remodeling." *Journal of Theoretical Biology* 262.2 (2010): 306–316. *ScienceDirect*. Web.

Pogoda, P. et al. "Bone Remodeling: New Aspects of a Key Process That Controls Skeletal Maintenance and Repair." *Osteoporosis international : a journal established as result of cooperation between the European Foundation for Osteoporosis and the National Osteoporosis Foundation of the USA* 16 Suppl 2.Journal Article (2005): S18-24. Web.

Scheiner, Stefan, Peter Pivonka, and Christian Hellmich. "Coupling Systems Biology with Multiscale Mechanics, for Computer Simulations of Bone Remodeling." *Computer Methods in Applied Mechanics and Engineering* 254.Journal Article (2013): 181–196. Web.

Sims, N. A., and J. H. Gooi. "Bone Remodeling: Multiple Cellular Interactions Required for Coupling of Bone Formation and Resorption." *Seminars in cell & developmental biology* 19.5 (2008): 444–451. Web.

Zhao, Y., Y. Cai, and Y. N. Wang. "RANKL and OPG Regulation on Bone Remodeling." *Shanghai kouqiangyixue = Shanghai journal of stomatology* 19.4 (2010): 443–446. Print.

Zumsande, Martin et al. "General Analysis of Mathematical Models for Bone Remodeling." *Bone* 48.4 (2011): 910–917. *ScienceDirect*. Web.

Macrophage lifespan

Chazaud, Bénédicte. "Macrophages: Supportive Cells for Tissue Repair and Regeneration." *Immunobiology* 219.3 (2014): 172–178. Print.

Cole, Joby et al. "Chapter Four - The Role of Macrophages in the Innate Immune Response to Streptococcus Pneumoniae and Staphylococcus Aureus: Mechanisms and Contrasts." *Advances in Bacterial Pathogen Biology* 65.Journal Article (2014): 125–202. Print.

Dancik, Garrett M., Douglas E. Jones, and Karin S. Dorman. "Parameter Estimation and Sensitivity Analysis in an Agent-Based Model of

Leishmania Major Infection." *Journal of Theoretical Biology* 262.3 (2010): 398–412. *ScienceDirect*. Web.

Dockrell, D. H., and M. K. Whyte. "Regulation of Phagocyte Lifespan in the Lung during Bacterial Infection." *Journal of leukocyte biology* 79.5 (2006): 904–908. Print.

Epelman, Slava, Kory J Lavine, and Gwendalyn J Randolph. "Origin and Functions of Tissue Macrophages." *Immunity* 41.1 (2014): 21–35. Print.

Mulherin, Diarmuid, Oliver Fitzgerald, and Barry Bresnihan. "Synovial Tissue Macrophage Populations and Articular Damage in Rheumatoid Arthritis." *Arthritis & Rheumatism* 39.1 (1996): 115–124. *Wiley Online Library*. Web.

Smith, Amber M., Jonathan A. McCullers, and Frederick R. Adler. "Mathematical Model of a Three-Stage Innate Immune Response to a Pneumococcal Lung Infection." *Journal of Theoretical Biology* 276.1 (2011): 106–116. *ScienceDirect*. Web.

Xaus, Jordi et al. "Molecular Mechanisms Involved in Macrophage Survival, Proliferation, Activation or Apoptosis." *Immunobiology* 204.5 (2001): 543–550. Print.

Monocyte lifespan

Brunet de la Grange, Philippe, et al. "Long-Term Repopulating Hematopoietic Stem Cells and 'Side Population' in Human Steady State Peripheral Blood." *Stem Cell Research* 11.1 (2013): 625–633. Print.

Dale, D. C., L. Boxer, and W. C. Liles. "The Phagocytes: Neutrophils and Monocytes." *Blood* 112.4 (2008): 935–945. Print.

Fehder, W. P. et al. "Macrophages*." *Encyclopedia of Stress (Second Edition)*. Ed. George Fink. Book, Section. New York: Academic Press, 2007. 634–639. Web.

Ginhoux, Florent, and Steffen Jung. "Monocytes and Macrophages: Developmental Pathways and Tissue Homeostasis." *Nature Reviews Immunology* 14.6 (2014): 392–404. *www.nature.com*. Web.

Goasguen, J. E. et al. "Morphological Evaluation of Monocytes and Their Precursors." *Haematologica* 94.7 (2009): 994–997. Print.

Gonzalez-Mejia, M. E., and A. I. Doseff. "Regulation of Monocytes and Macrophages Cell Fate." *Frontiers in bioscience (Landmark edition)*14.Journal Article (2009): 2413–2431. Print.

Italiani, Paola, and Diana Boraschi. "From Monocytes to M1/M2 Macrophages: Phenotypical vs. Functional Differentiation." *Frontiers in Immunology* 5 (2014): n. pag. *PubMed Central*. Web. 10 Aug. 2017.

Parihar, A., T. D. Eubank, and A. I. Doseff. "Monocytes and Macrophages Regulate Immunity through Dynamic Networks of Survival and Cell Death." *Journal of innate immunity* 2.3 (2010): 204–215. Print.

Patel, A. A. et al. "The Fate and Lifespan of Human Monocyte Subsets in Steady State and Systemic Inflammation." *The Journal of experimental medicine* 214.7 (2017): 1913–1923. Print.

Reuter, S., and D. Lang. "Life Span of Monocytes and Platelets: Importance of Interactions." *Frontiers in bioscience (Landmark edition)*14.Journal Article (2009): 2432–2447. Print.

Whitelaw, D. M., and Maureen Bell. "The Intravascular Lifespan of Monocytes." *Blood* 28.3 (1966): 455–464. Print.

Yona, S. et al. "Fate Mapping Reveals Origins and Dynamics of Monocytes and Tissue Macrophages under Homeostasis." *Immunity* 38.1 (2013): 79–91. Print.

Neutrophil lifespan

Anwar, S., and M. K. Whyte. "Neutrophil Apoptosis in Infectious Disease." *Experimental lung research* 33.10 (2007): 519–528. Print.

Bekkering S (2013) Another look at the life of a neutrophil. *World Journal of Hematology* 2:44.

Kaplanski, Gilles et al. "IL-6: A Regulator of the Transition from Neutrophil to Monocyte Recruitment during Inflammation." *Trends in Immunology*24.1 (2003): 25–29. *ScienceDirect*. Web.

Kettritz, R. et al. "Extracellular Matrix Regulates Apoptosis in Human Neutrophils." *Kidney international* 55.2 (1999): 562–571. Web.

Kumar, V., and A. Sharma. "Neutrophils: Cinderella of Innate Immune System." *International immunopharmacology* 10.11 (2010): 1325–1334. Web.

Maianski, N. A. et al. “Apoptosis of Neutrophils.” *Acta Haematologica* 111.1–2 (2004): 56–66. Web.

Rankin, S. M. “The Bone Marrow: A Site of Neutrophil Clearance.” *Journal of leukocyte biology* 88.2 (2010): 241–251. Web.

Schröder, J. -M. “Chemoattractants as Mediators of Neutrophilic Tissue Recruitment.” *Clinics in dermatology* 18.3 (2000): 245–263. Web.

Smith, Amber M., Jonathan A. McCullers, and Frederick R. Adler. “Mathematical Model of a Three-Stage Innate Immune Response to a Pneumococcal Lung Infection.” *Journal of theoretical biology* 276.1 (2011): 106–116. Web.

Summers, C. et al. “Neutrophil Kinetics in Health and Disease.” *Trends in immunology* 31.8 (2010): 318–324. Web.

Whyte, M. et al. “Apoptosis and the Regulation of Neutrophil Lifespan.” *Biochemical Society transactions* 27.6 (1999): 802–807. Print.

TGF

Ashcroft, Gillian S. “Bidirectional Regulation of Macrophage Function by TGF-β.” *Microbes and Infection* 1.15 (1999): 1275–1282. *ScienceDirect*. Web.

Bismar, H et al. “Transforming Growth Factor β (TGF-β) Levels in the Conditioned Media of Human Bone Cells: Relationship to Donor Age, Bone Volume, and Concentration of TGF-β in Human Bone Matrix in Vivo.” *Bone* 24.6 (1999): 565–569. *ScienceDirect*. Web.

Kelly, Aoife et al. “Chapter Four - Regulation of Innate and Adaptive Immunity by TGFβ.” *Advances in Immunology* 134.Journal Article (2017): 137–233. Print.

Pivonka, Peter et al. “Model Structure and Control of Bone Remodeling: A Theoretical Study.” *Bone* 43.2 (2008): 249–263. *ScienceDirect*. Web.

Schmidt-Weber, Carsten B., and Kurt Blaser. “Regulation and Role of Transforming Growth Factor-β in Immune Tolerance Induction and Inflammation.” *Current opinion in immunology* 16.6 (2004): 709–716. Print.

Sheng, J., W. Chen, and H. J. Zhu. “The Immune Suppressive Function of Transforming Growth Factor-Beta (TGF-Beta) in Human Diseases.” *Growth factors (Chur, Switzerland)* 33.2 (2015): 92–101. Print.

Wahl, Sharon M. “Transforming Growth Factor-β: Innately Bipolar.” *Innate immunity/Antigen processing and recognition* 19.1 (2007): 55–62. Print.

Immune system mathematical or computational model

Afacan, Nicole J., Christopher D. Fjell, and Robert E. W. Hancock. “A Systems Biology Approach to Nutritional Immunology – Focus on Innate Immunity.” *Nutritional Immunology* 33.1 (2012): 14–25. Print.

Herald, M. C. “General Model of Inflammation.” *Bulletin of mathematical biology* 72.4 (2010): 765–779. Print.

Kalita, J. K. et al. “Computational Modelling and Simulation of the Immune System.” *International journal of bioinformatics research and applications* 2.1 (2006): 63–88. Print.

Lehnert, T. et al. “Bottom-up Modeling Approach for the Quantitative Estimation of Parameters in Pathogen-Host Interactions.” *Frontiers in microbiology*6.Journal Article (2015): 608. Print.

Marino, S., E. Beretta, and D. E. Kirschner. “The Role of Delays in Innate and Adaptive Immunity to Intracellular Bacterial Infection.” *Mathematical biosciences and engineering : MBE* 4.2 (2007): 261–288. Print.

Nakaoka, Shinji. “Mathematical Modeling and Simulation for a Coupled Communication System of Immune Cells*.” *4th IFAC Conference on Analysis and Control of Chaotic Systems CHAOS 2015Tokyo, Japan, 26–28 August 2015* 48.18 (2015): 41–46. Print.

Tegner, J. et al. “Systems Biology of Innate Immunity.” *Cellular immunology* 244.2 (2006): 105–109. Print.

Woelke, Anna Lena, Manuela S. Murgueitio, and Robert Preissner. “Theoretical Modeling Techniques and Their Impact on Tumor Immunology.” *Clinical and Developmental Immunology* 2010 (2010): n. pag. *PubMed Central*. Web. 11 Aug. 2017.

Wolf, Katarina, and Peter Friedl. “Extracellular Matrix Determinants of Proteolytic and Non-Proteolytic Cell Migration.” *Trends in cell biology* 21.12 (2011): 736–744. Print.

Zhang, Yue, and James B. Bliska. “Mathematical Relationship between Cytokine Concentrations and Pathogen Levels during Infection.” *Cytokine* 53.2 (2011): 158–162. Print.

Bacteria count

Gaudin, A. et al. “A New Experimental Model of Acute Osteomyelitis Due to Methicillin-Resistant Staphylococcus Aureus in Rabbit.” *Letters in applied microbiology* 52.3 (2011): 253–257. Web.

Jarrett, A. M., N. G. Cogan, and M. E. Shirtliff. “Modelling the Interaction between the Host Immune Response, Bacterial Dynamics and Inflammatory Damage in Comparison with Immunomodulation and Vaccination Experiments.” *Mathematical medicine and biology : a journal of the IMA* 32.3 (2015): 285–306. Web.

Paharik, Alexandra E., and Alexander R. Horswill. “The Staphylococcal Biofilm: Adhesins, Regulation, and Host Response.” *Microbiology Spectrum* 4.2 (2016): n. pag. *www.asmscience.org*. Web.

Søe, Niels H. et al. “A Novel Knee Prosthesis Model of Implant-Related Osteo- Myelitis in Rats.” *Acta Orthopaedica* 84.1 (2013): 92–97. *Taylor and Francis+NEJM*. Web.

Bacteria production rate

Ansari, S. et al. “Staphylococcus Aureus: Methicillin Resistance and Small Colony Variants from Pyogenic Infections of Skin, Soft Tissue and Bone.” *Journal of Nepal Health Research Council* 13.30 (2015): 126–132. Print.

Cramton, Sarah E., Christiane Gerke, and Friedrich Götz. “In Vitro Methods to Study Staphylococcal Biofilm Formation.” *Microbial Growth in Biofilms - Part A: Developmental and Molecular Biological Aspects* 336.Journal Article (2001): 239–255. Web.

DosReis, George A., and Marcello A. Barcinski. “Apoptosis and Parasitism: From the Parasite to the Host Immune Response.” *Advances in Parasitology*49.Journal Article (2001): 133–161. Web.

Li, D. et al. “Quantitative Mouse Model of Implant-Associated Osteomyelitis and the Kinetics of Microbial Growth, Osteolysis, and Humoral Immunity.” *Journal of orthopaedicresearch : official publication of the Orthopaedic Research Society* 26.1 (2008): 96–105. Web.

Melter, O., and B. Radojevic. “Small Colony Variants of Staphylococcus Aureus--Review.” *Folia microbiologica* 55.6 (2010): 548–558. Web.

Moss, Jeremy E., Antonios O. Aliprantis, and Arturo Zychlinsky. “The Regulation of Apoptosis by Microbial Pathogens.” *International review of cytology* 187.Journal Article (1999): 203–259. Web.

Widaa, A. et al. “Staphylococcus Aureus Protein A Plays a Critical Role in Mediating Bone Destruction and Bone Loss in Osteomyelitis.” *PloS one* 7.7 (2012): e40586. Web.

BJI computational model

Lio, P. et al. “Modelling Osteomyelitis.” *BMC bioinformatics* 13 Suppl14.Journal Article (2012): S12–2105–13–S14–S12. Epub 2012 Sep 7. Web.

Interaction Bacteria and Bone cells

Akdis, Mübeccel et al. “Interleukins (from IL-1 to IL-38), Interferons, Transforming Growth Factor β, and TNF-α: Receptors, Functions, and Roles in Diseases.” *Journal of Allergy and Clinical Immunology* 138.4 (2016): 984–1010. Web.

Bost, K. L. et al. “Staphylococcus Aureus Infection of Mouse or Human Osteoblasts Induces High Levels of Interleukin-6 and Interleukin-12 Production.” *The Journal of infectious diseases* 180.6 (1999): 1912–1920. Print.

Chen, Q. et al. “Involvement of Toll-like Receptor 2 and pro-Apoptotic Signaling Pathways in Bone Remodeling in Osteomyelitis.” *Cellular physiology and biochemistry : international journal of experimental cellular physiology, biochemistry, and pharmacology* 34.6 (2014): 1890–1900. Web.

Claro, T. et al. “Staphylococcus Aureus Protein A Binding to Osteoblast Tumour Necrosis Factor Receptor 1 Results in Activation of Nuclear Factor Kappa B and Release of Interleukin-6 in Bone Infection.” *Microbiology (Reading, England)* 159.Pt 1 (2013): 147–154. Web.

Ellington, John K. et al. “Mechanisms Of Staphylococcus Aureus invasion of Cultured Osteoblasts.” *Microbial Pathogenesis* 26.6 (1999): 317–323. *ScienceDirect*. Web.

Josse, J., F. Velard, and S. C. Gangloff. “Staphylococcus Aureus vs. Osteoblast: Relationship and Consequences in Osteomyelitis.” *Frontiers in cellular and infection microbiology*5.Journal Article (2015): 85. Web.

Junka, A. et al. “Bad to the Bone: On In Vitro and Ex Vivo Microbial Biofilm Ability to Directly Destroy Colonized Bone Surfaces without Participation of Host Immunity or Osteoclastogenesis.” *PloS one* 12.1 (2017): e0169565. Web.

Marriott, I. “Apoptosis-Associated Uncoupling of Bone Formation and Resorption in Osteomyelitis.” *Frontiers in cellular and infection microbiology*3.Journal Article (2013): 101. Web.

---. “Osteoblast Responses to Bacterial Pathogens: A Previously Unappreciated Role for Bone-Forming Cells in Host Defense and Disease

Progression." *Immunologic research* 30.3 (2004): 291–308. Print.

---. "Osteoblasts Express the Inflammatory Cytokine Interleukin-6 in a Murine Model of Staphylococcus Aureus Osteomyelitis and Infected Human Bone Tissue." *The American journal of pathology* 164.4 (2004): 1399–1406. Print.

Interaction bacteria and immune cells

Asensi, V. et al. "In Vivo Interleukin-6 Protects Neutrophils from Apoptosis in Osteomyelitis." *Infection and immunity* 72.7 (2004): 3823–3828. Web.

Carmichael, Andrew, and Mark Wills. "The Immunology of Infection." *Infections Part 1 of 3* 41.11 (2013): 611–618. Web.

Kim, Hwan Keun et al. "Recurrent Infections and Immune Evasion Strategies of Staphylococcus Aureus." *Host—microbe interactions: bacteria* 15.1 (2012): 92–99. Web.

Ning, R. et al. "Staphylococcus Aureus Regulates Secretion of Interleukin-6 and Monocyte Chemoattractant Protein-1 through Activation of Nuclear Factor KappaB Signaling Pathway in Human Osteoblasts." *The Brazilian journal of infectious diseases : an official publication of the Brazilian Society of Infectious Diseases* 15.3 (2011): 189–194. Print.

Sottnik, J. L. et al. "Chronic Bacterial Osteomyelitis Suppression of Tumor Growth Requires Innate Immune Responses." *Cancer immunology, immunotherapy : CII* 59.3 (2010): 367–378. Web.

Interaction bacteria and signals

Aggarwal, Bharat B. "Nuclear Factor-KB: The Enemy Within." *Cancer Cell* 6.3 (2004): 203–208. Web.

Cassat, J. E. et al. "A Secreted Bacterial Protease Tailors the Staphylococcus Aureus Virulence Repertoire to Modulate Bone Remodeling during Osteomyelitis." *Cell host & microbe* 13.6 (2013): 759–772. Web.

Ning, R. et al. "Attachment of Staphylococcus Aureus Is Required for Activation of Nuclear Factor Kappa B in Human Osteoblasts." *Acta biochimica et biophysicaSinica* 42.12 (2010): 883–892. Web.

Ning, R. D. et al. "Activation of Nuclear Factor KappaB Signaling Pathway in Human Osteoblasts Responses to Staphylococcus Aureus in Vitro." *Zhonghuawaikezazhi [Chinese journal of surgery]* 50.3 (2012): 264–267. Print.

Sharma, Rakesh, and Stefan D. Anker. "Cytokines, Apoptosis and Cachexia: The Potential for TNF Antagonism." *Cachexia* 85.1 (2002): 161–171. Web.

Szondy, Zsuzsa, and Anna Pallai. "Transmembrane TNF-Alpha Reverse Signaling Leading to TGF-Beta Production Is Selectively Activated by TNF Targeting Molecules: Therapeutic Implications." *Pharmacological Research* 115.Journal Article (2017): 124–132. Web.

Young, A. B. et al. "Causative Agents of Osteomyelitis Induce Death Domain-Containing TNF-Related Apoptosis-Inducing Ligand Receptor Expression on Osteoblasts." *Bone* 48.4 (2011): 857–863. Web.

Zhu, Yuwen, Sheng Yao, and Lieping Chen. "Cell Surface Signaling Molecules in the Control of Immune Responses: A Tide Model." *Immunity* 34.4 (2011): 466–478. Web.

Interaction bone cells and immune cells

Allaeys, Isabelle et al. "Osteoblast Retraction Induced by Adherent Neutrophils Promotes Osteoclast Bone Resorption: Implication for Altered Bone Remodeling in Chronic Gout." *Laboratory Investigation* 91.6 (2011): 905–920. *www.nature.com.scd-proxy.univ-brest.fr*. Web.

El-Jawhari, Jehan J., Elena Jones, and Peter V. Giannoudis. "The Roles of Immune Cells in Bone Healing; What We Know, Do Not Know and Future Perspectives." *Injury* 47.11 (2016): 2399–2406. Web.

Ferrari-Lacraz, S., and S. Ferrari. "Do RANKL Inhibitors (Denosumab) Affect Inflammation and Immunity?" *Osteoporosis international : a journal established as result of cooperation between the European Foundation for Osteoporosis and the National Osteoporosis Foundation of the USA* 22.2 (2011): 435–446. Web.

Michalski, Megan N., and Laurie K. McCauley. "Macrophages and Skeletal Health." *Pharmacology & therapeutics* 174.Journal Article (2017): 43–54. Web.

Nakashima, Tomoki, and Hiroshi Takayanagi. "The Dynamic Interplay between Osteoclasts and the Immune System." *Archives of Biochemistry and Biophysics* 473.2 (2008): 166–171. *ScienceDirect*. Web. Highlight Issue: Bone Remodeling: Facts and Perspectives.

Takayanagi, Hiroshi. "Osteoimmunology: Shared Mechanisms and Crosstalk between the Immune and Bone Systems." *Nature Reviews Immunology* 7.4 (2007): 292–304. *www.nature.com.scd-proxy.univ-brest.fr*. Web.
